# Narrowing down the targets for yield improvement in rice under normal and abiotic stress conditions via expression profiling of yield-related genes

**DOI:** 10.1186/1939-8433-5-37

**Published:** 2012-12-22

**Authors:** Amit K Tripathi, Ashwani Pareek, Sudhir K Sopory, Sneh L Singla-Pareek

**Affiliations:** Plant Molecular Biology, International Centre for Genetic Engineering and Biotechnology, Aruna Asaf Ali Road, New Delhi, 110067 India; Stress Physiology and Molecular Biology Laboratory, School of Life Sciences, Jawaharlal Nehru University, New Delhi, 110067 India

**Keywords:** Rice, Grain yield, Development, Yield penalty, Environmental stresses, Microarray, qRT-PCR

## Abstract

**Background:**

Crop improvement targeting high yield and tolerance to environmental stresses has become the need of the hour. Yield improvement via breeding or gene pyramiding aiming comprehensive incorporation of the agronomically favored traits requires an in-depth understanding of the molecular basis of these traits. The present study describes expression profiling of yield-related genes in rice with respect to different developmental stages and various abiotic stress conditions.

**Results:**

Our analysis indicates developmental regulation of the yield-related genes pertaining to the genetic reprogramming involved at the corresponding developmental stage. The gene expression data can be utilized to specifically select particular genes which can potentially function synergistically for enhancing the yield while maintaining the source-sink balance. Furthermore, to gain some insights into the molecular basis of yield penalty during various abiotic stresses, the expression of selected yield-related genes has also been analyzed by qRT-PCR under such stress conditions. Our analysis clearly showed a tight transcriptional regulation of a few of these yield-related genes by abiotic stresses. The stress-responsive expression patterns of these genes could explain some of the most important stress-related physiological manifestations such as reduced tillering, smaller panicles and early completion of the life cycle owing to reduced duration of vegetative and reproductive phases.

**Conclusions:**

Development of high yielding rice varieties which maintain their yield even under stress conditions may be achieved by simultaneous genetic manipulation of certain combination of genes such as *LRK1* and *LOG*, based on their function and expression profile obtained in the present study. Our study would aid in investigating in future, whether over-expressing or knocking down such yield-related genes can improve the grain yield potential in rice.

**Electronic supplementary material:**

The online version of this article (doi:10.1186/1939-8433-5-37) contains supplementary material, which is available to authorized users.

## Background

Rice is one of the most important staple food crops feeding almost half of the world population. There are many reasons for the growing concern about sustainable and sufficient production of various staple food crops including rice. Some of these are the ever-increasing population, less availability of arable land, global climate changes and decreasing availability of water for agriculture (Takeda and Matsuoka, 2008). In order to support the growing human population, a more sustainable means of rice production is needed. Specifically, crop improvement targeting high-yield and enhanced tolerance to various environmental stresses has become an urgent necessity.

Being a complex trait, grain yield in rice is determined by the three component traits viz. grain number per panicle, grain weight, and number of panicles per plant (Xing and Zhang, 2010). Number of grains per panicle further depends on number of spikelets; which is primarily determined by the degree of branching; and seed setting rate of the spikelets. The sub-component traits affecting grain weight are the three-dimensional size of the grains and the ratio of filled grains. Number of panicles per plant depends on the tillering ability of the plant. In addition, some traits like plant height indirectly determine the yield potential by affecting light capture by photosynthesis (Sakamoto et al. 2006) or lodging (Khush, 1999).

Rice yield is considered to be a quantitative trait controlled by multiple genes, each contributing in a small but significant way. Quantitative trait loci (QTL) mapping has led to the prediction of several QTLs for yield-related traits (http://www.gramene.org/db/qtl/qtl_display?trait_category=Yield). The development of techniques for QTL validation and analysis has helped in deciphering the genetic basis of yield traits. Coordinated efforts in rice functional genomics, owing to the completion of rice genome sequencing, have led to the identification of several genes, corresponding to such QTLs (Miura et al. 2011). There are altogether twenty-three genes reported, till date, to contribute to one or the other yield-related traits viz. tiller number, panicle development, grain number and grain size (see Table1). Such knowledge of molecular basis of grain yield in rice can be exploited in yield improvement programs via gene pyramiding and/or the breeding approaches. However, considering the complexity of the yield traits, a limitation of these approaches is that not a large number of genes can be beneficially engineered simultaneously, thus making it essential to select few genes which can function synergistically in order to get the desired outcome. Moreover, while engineering for yield improvement, the source-sink balance in plants must be taken into consideration to avoid deleterious effects. In order to achieve the above objective, we need to extensively assess the expression patterns of the genes regulating yield traits.

**Table 1:** **Functional classification of yield-related genes in rice**

Trait Regulated	Sub-component trait	Name of the gene	Locus Id	Predicted gene product as per RGAP 7 (Ouyang et al.^2007^)	References
Grain number per panicle	Panicle development	*LAX1*	LOC_Os01g61480	helix-loop-helix DNA-binding domain containing protein	Komatsu et al. 2003a; Oikawa and Kyozuka, 2009
		*OsSPL14*	LOC_Os08g39890	OsSPL14 - SBP-box gene family member	Miura et al. 2010
		*FZP*	LOC_Os07g47330	AP2 domain containing protein	Komatsu et al. 2003b
	Rate of spikelet formation	*DEP1*	LOC_Os09g26999	keratin-associated protein 5-4	Huang et al. 2009
		*SP1*	LOC_Os11g12740	peptide transporter PTR2	Li et al. 2009
		*APO1*	LOC_Os06g45460	OsFBX202 - F-box domain containing protein	Ikeda-Kawakatsu et al. 2009
		*LOG*	LOC_Os01g40630	uncharacterized protein PA4923	Kurakawa et al. 2007
		*OsCKX2*	LOC_Os01g10110	cytokinin dehydrogenase	Ashikari et al. 2005
	Duration of panicle differentiation	*RCN1*	LOC_Os11g05470	Phosphatidylethanolamine-binding protein	Nakagawa et al. 2002
		*RFL*	LOC_Os04g51000	transcription factor FL	Rao et al. 2008
		*Ghd7*	LOC_Os07g15770	CCT motif family protein	Xue et al. 2008
Tillering		*LRK1*	LOC_Os02g05980	phytosulfokine receptor	Zha et al. 2009
		*OsTB1*	LOC_Os03g49880	TCP family transcription factor	Takeda et al. 2003
		*D10*	LOC_Os01g54270	transposon protein	Arite et al. 2007
		*Htd1*	LOC_Os04g46470	carotenoid cleavage dioxygenase 7	Zou et al. 2006
		*D3*	LOC_Os06g06050	F-box domain and LRR containing protein	Ishikawa et al. 2005
		*MOC1*	LOC_Os06g40780	Monoculm 1	Li et al. 2003
Grain weight	Grain width	*GW2*	LOC_Os02g14720	expressed protein	Song et al. 2007
	Grain length and size	*GS3*	LOC_Os03g29380	conserved hypothetical protein	Fan et al. 2006
	Grain filling	*GIF1*	LOC_Os04g33740	glycosyl hydrolase	Wang et al. 2008
Plant Height		*Sd1*	LOC_Os01g66100	gibberellin 20 oxidase 2	Sasaki et al. 2002
		*OsBRI1*	LOC_Os01g52050	systemin receptor SR160 precursor	Morinaka et al. 2006
		*OsEATB*	LOC_Os09g28440	AP2 domain containing protein	Qi et al. 2011

Remarkable difference in grain yield is found among different rice genotypes with variability in the combinations of the component traits. Besides, grain yield of different rice cultivars is greatly influenced by the prevailing environmental conditions and farming practices. Indeed, one of the most serious impacts of abiotic stresses is ‘yield penalty’ i.e. reduction in grain yield (Hirayama and Shinozaki, 2010; Urano et al. 2010). The reasons for this severe reduction in yield under abiotic stress conditions include improper growth, early senescence, reduced photosynthesis, less tillering, reduced panicle branching, and inadequate grain filling. However, the molecular basis of this ‘yield penalty’ is still not well understood.

The present study describes a microarray-based expression profiling of a set of specific genes, reported to regulate yield-related traits in rice, with respect to the major developmental stages of rice. These physiologically distinct developmental stages are germination, seedling, tillering, stem elongation, booting, heading, flowering, milk, and dough. In addition, to gain insights into the molecular basis of yield penalty during various abiotic stresses, expression of the said genes has also been analyzed using publicly available microarray data and further validated by quantitative RT-PCR (qRT-PCR). To attribute a basis to the observed differential expression of some of the yield-related genes under different abiotic stress conditions, putative cis-regulatory elements present in the upstream promoter region of these genes have also been predicted *in silico*.

## Results

**Figure 1:**
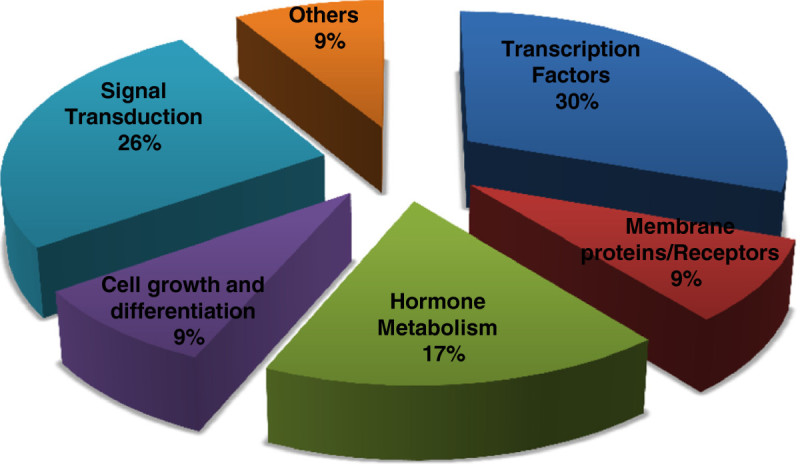
**Classification of genes regulating yield-traits.** Pie-chart showing distribution of the yield-related genes in various classes on the basis of cellular functions predicted for the encoded proteins as per RGAP7 – Rice genome database. As is evident from the chart, more than half of the genes belong either to the class of transcription factors or signaling proteins.

Based on their predicted function in various previous studies, we classified the twenty-three genes reported to regulate yield traits in rice into four major categories as (1) genes controlling number of grains per panicle, (2) genes regulating number of tillers, (3) genes for grain weight, and (4) genes controlling plant height. These genes in each category have been documented to have their role in affecting different sub-component traits such as panicle development, rate of spikelet formation, duration of panicle differentiation, grain width, grain length and size, grain filling etc. (Table1). On the basis of cellular functions predicted for the encoded proteins, these genes can be categorized into various functional classes (Figure1) such as transcription factors (30%), membrane proteins/receptors (9%), or those involved in signal transduction (26%), hormone metabolism (17%), cell growth, and differentiation (9%).

The microarray-based expression data of these genes during development and abiotic stress conditions were retrieved, curated, normalized and analyzed with the help of publicly available database tool Genevestigator (Hruz et al. 2008) using default parameters. Furthermore, qRT-PCR based expression analysis was carried out for those genes that showed significant differential expression (in microarray-based expression analysis) under different abiotic stress conditions.

### Developmental regulation of genes controlling grain number per panicle

**Figure 2:**
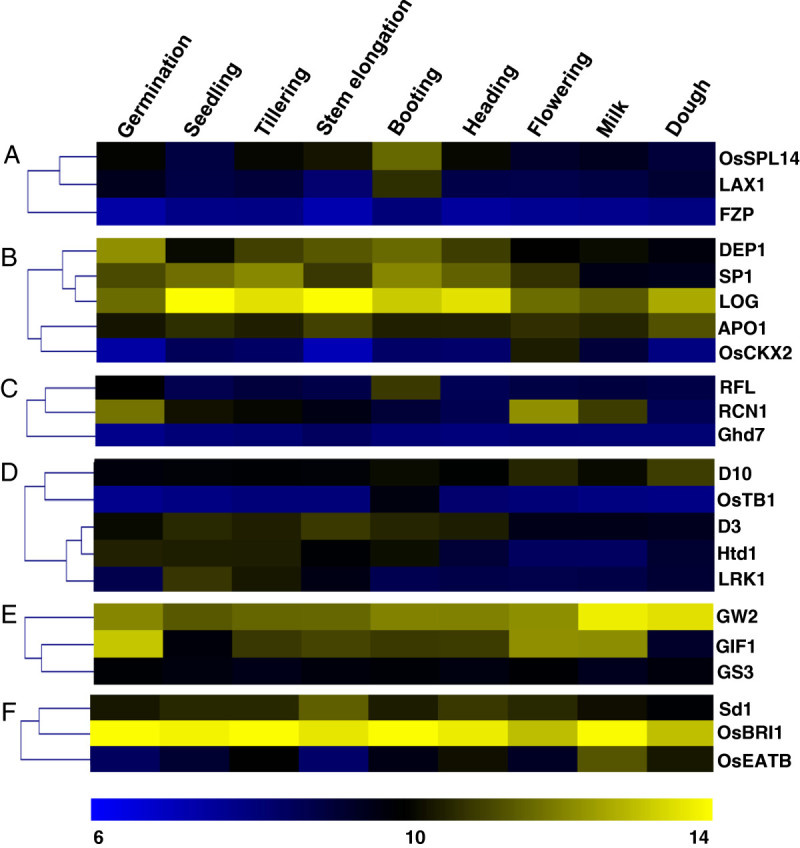
**Developmental expression profile of various functional classes of yield-related genes.** Heat maps show microarray-based developmental expression profile based on hierarchical clustering of different classes of yield-related genes viz. genes controlling, **(A)** panicle development, **(B)** rate of spikelet formation, **(C)** duration of panicle differentiation, **(D)** Tillering, **(E)** grain weight, and **(F)** plant height. The nine physiologically distinct developmental stages in which expression of these genes was analyzed are – germination, seedling, tillering, stem elongation, booting, heading, flowering, milk, and dough; as shown at the top of the heat map. The heat maps were generated using values for log_2_-transformed mean signal intensities on Affymetrix 51 K array for each of the genes in respective developmental stages. Clustering in the heat maps is based on average linkage method and Pearson correlation distance metric. Color bar at the bottom represents scale for log_2_ expression (signal intensity).

One of the major traits which determine the overall yield is the number of grains per panicle which is controlled by genes regulating panicle development, or the rate of spikelet formation or the duration of panicle differentiation (Table1). Panicle development has been shown to be controlled by the action of either of the three genes viz. *LAX1* (Komatsu et al. 2003a; Oikawa and Kyozuka, 2009); *OsSPL14* (Miura et al. 2010) and *FZP* (Komatsu et al. 2003b). While *LAX1* and *OsSPL14* serve as the positive regulators of panicle development, *FZP* is required for maintaining floral meristem identity. In our analysis, we found that the expression of both *LAX1* and *OsSPL14* significantly increased at the booting stage (Figure2A). This confirms their predicted role in panicle branching, as reported earlier (Oikawa and Kyozuka, 2009; Miura et al. 2010). Further, we observed the expression of *FZP* gene to be uniformly low throughout the development stages analyzed here.

Genes reported to regulate rate of spikelet formation are *DEP1* (Huang et al. 2009), *SP1* (Li et al. 2009), *APO1* (Ikeda-Kawakatsu et al. 2009), *LOG* (Kurakawa et al. 2007), and *OsCKX2* (Ashikari et al. 2005). All, but *OsCKX2*, are known to enhance the rate of spikelet formation. Among the positive regulators, DEP1 was highly expressed from the tillering to the heading stage while the expression of *SP1* was higher until the flowering stage (Figure2B). This suggests that these genes affect the meristematic activity and cell proliferation. Further, we found high expression of *LOG* gene throughout the plant development (Figure2B). *LOG* is predicted to encode an uncharacterized protein PA4923 (Table1) in the RGAP7 rice genome browser (Ouyang et al. 2007); however a study by Kurakawa et al. (2007) has found the product of this gene to be a lysine decarboxylase. The expression pattern of *LOG* obtained here is consistent to its predicted role in maintaining meristem activity (Kurakawa et al. 2007). The negative regulator *OsCKX2*, coding for an enzyme cytokinin oxidase/dehydrogenase involved in cytokinin metabolism, had low expression levels until the heading stage. Further, we found an inverse-correlation between the expression pattern of *LOG* and *CKX2*, in most of the developmental stages analyzed here (Figure2B). The other positive regulator *APO1* maintained fairly constant expression levels during development (Figure2B).

Amongst the genes determining the duration of panicle differentiation, the highest expression of the gene *RCN1* which codes for a putative PEBP (Nakagawa et al. 2002), was at the flowering stage (Figure2C). Besides, we found that the expression of *RFL* gene was significantly higher at the booting stage (Figure2C); suggesting its role in vegetative to flowering stage transition. In our analysis, the expression of *Ghd7,* reported to regulate photoperiodic flowering (Xue et al. 2008), did not show significant alterations in its transcript levels over different developmental stages (Figure2C).

### Developmental regulation of genes affecting tillering process

Number of tillers determine the number of panicles per plant and hence the number of grains. Tillering requires the fine-tuning of expression of many genes such as *MOC1* (Li et al. 2003), *LRK1* (Zha et al. 2009), *OsTB1* (Takeda et al. 2003), *D10* (Arite et al. 2007), *Htd1* (Zou et al. 2006), and *D3* (Ishikawa et al. 2005). Amongst these, *MOC1* and *LRK1* promote the formation of tillers whilst others function as negative regulators. In our analysis of the expression of the above genes, we found that *LRK1* is expressed more at the seedling and tillering stages; whereas *OsTB1* had very low expression level at these stages (Figure2D). In case of other negative regulators, the expression data could not explain any defined pattern of regulation (Figure2D).

### Developmental expression profile of genes regulating grain weight

The parameters determining grain weight are grain length, width and thickness, besides grain filling. The genes reported to regulate these parameters are *GW2*, *GS3*, and *GIF1*. *GW2* encodes a RING-type ubiquitin E3 ligase and previous studies have shown that *GW2* negatively regulates grain width (Song et al. 2007). We found that its highest expression levels are at the ‘milk’ and ‘dough’ stages (Figure2E); leading to the slender grain phenotype found in elite cultivars. For another negative regulator of grain length and size – *GS3,* we found lower expression levels throughout the stages of development (Figure2E). The other gene known to positively regulate grain filling, GIF1 (Wang et al. 2008), had higher expression in the flowering and milk stages of the reproductive phase.

### Developmental regulation of genes regulating plant height

An indirect determinant of yield is plant height; as dwarf plants are known to have higher productivity (Sakamoto and Matsuoka, 2008). One of the genes that positively regulates plant height is the green revolution gene *Sd1,* coding for an enzyme involved in gibberellin biosynthesis – gibberellin 20 oxidase 2 (Sasaki et al. 2002). We found *Sd1* to be expressed more at the vegetative stages with its expression peak at stem elongation stage (Figure2F), owing to the requirement of gibberellin at these stages. The other gene which partly regulates plant height positively is *OsBRI1,* encoding a receptor for the hormone brassinosteroid (Morinaka et al. 2006). We observed an interesting pattern of expression of this gene (Figure2F) with its fairly high expression in both vegetative and reproductive phases of development. While its high expression in the vegetative phase indicates its contribution to the usual ‘tall’ phenotype, its high expression in the reproductive stages is in consequence of the need of action of brassinosteroid hormones at this stage. The other gene known to negatively regulate plant height, *OsEATB* (Qi et al. 2011), had lower expression levels throughout the vegetative phase (Figure2F).

### Expression profiling of yield--related genes under various environmental stresses through microarray and qRT-PCR

**Figure 3:**
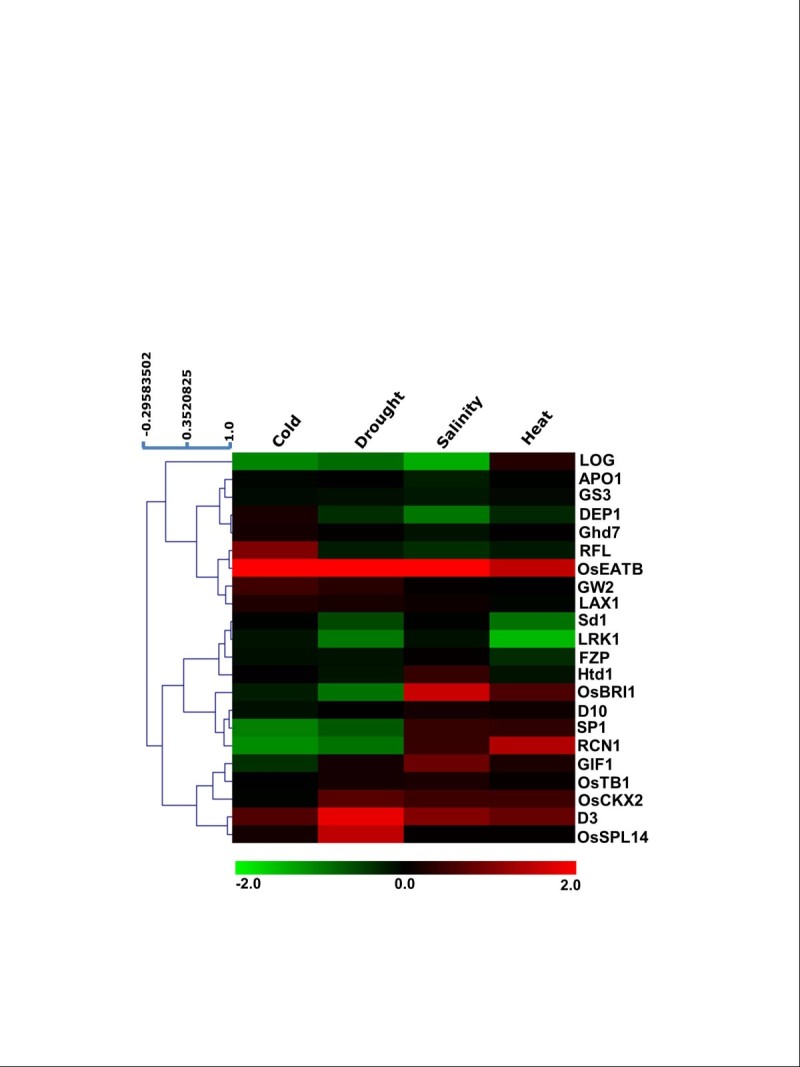
**Microarray-based expression profile of yield-related genes under different abiotic stress conditions.** Heat map shows expression profile based on hierarchical clustering of various yield-related genes under different stress-conditions viz. cold, drought, salt, and heat. Color bar at the bottom represents scale for log_2_ fold change in expression. For hierarchical clustering in the heat map, weighted average linkage method using Pearson correlation as the distance metric (scale shown at the top left of the heat map) was used. Eight of the genes viz. *D3*, *LRK1*, *OsEATB*, *RCN1*, *LOG*, *DEP1*, *SP1*, and *OsSPL14* were found to be significantly regulated in one or more abiotic stresses.

**Figure 4:**
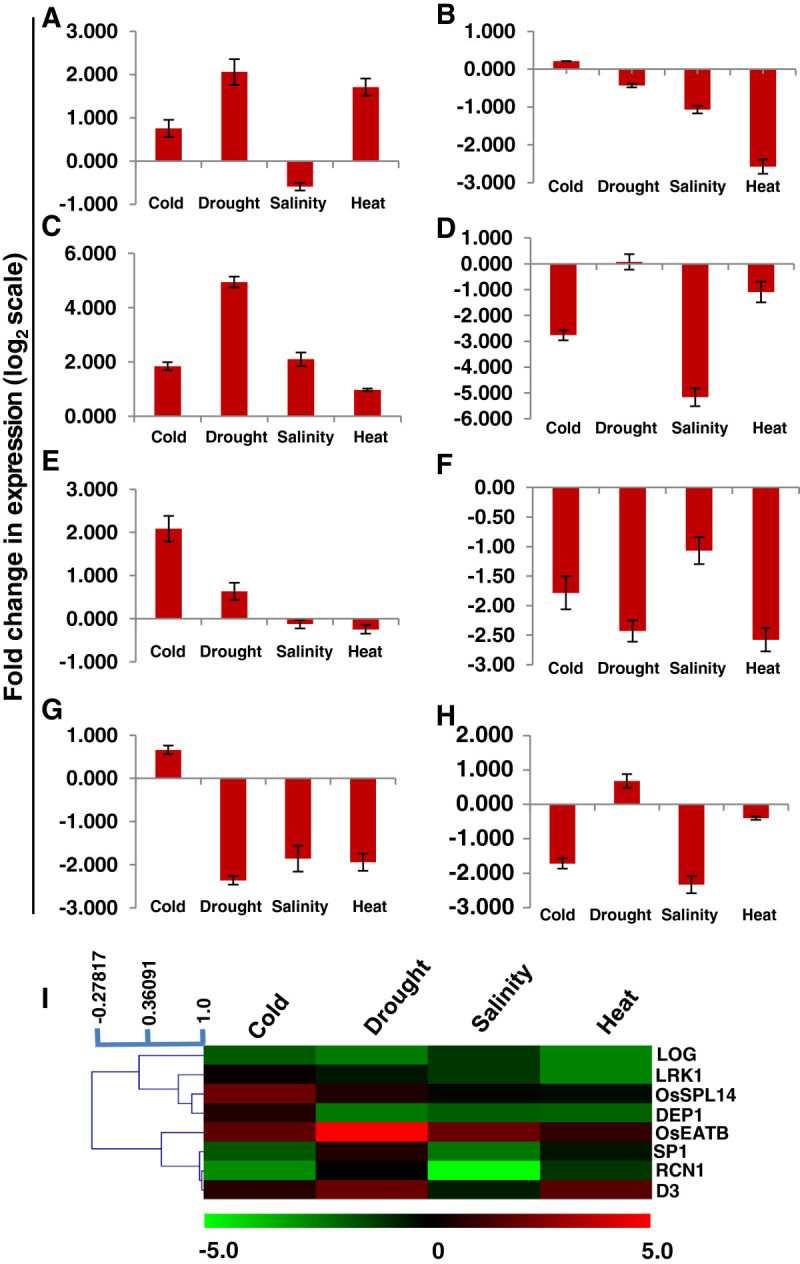
**qRT-PCR confirms altered expression of few yield-related genes under abiotic stress conditions.** Histograms **(A-H)** depict fold change (log_2_ scale) in expression of stress-regulated yield-related genes viz. **(A)** *D3*, **(B)** *LRK1*, **(C)** *OsEATB*, **(D)** *RCN1*, **(E)** *OsSPL14*, **(F)** *LOG*, **(G)** *DEP1*, **(H)** *SP1*; under different abiotic stress conditions – cold, drought, salinity, and heat as obtained via qRT-PCR. For expression analysis by qRT-PCR, 10 day old seedlings of IR64 variety (a moderately sensitive cultivar) of rice were subjected to stress treatment for 6 hours followed by RNA isolation, first strand cDNA synthesis and real-time PCR. Error bars show standard deviation. **(I)** Heat map generated on the basis of above changes in gene expression using average linkage hierarchical clustering with Pearson correlation as the distance metric. Color bar represents scale for log_2_ fold change in expression.

Next, we tried to gain insights into the molecular basis of ‘yield penalty’ through abiotic stress induced regulation of expression of the genes controlling yield traits, using publicly available microarrays. For this analysis, if fold change in expression in log_2_ scale (log_2_fc) ≥ 1.0 and *p-value* 0.05, then the gene was considered to be upregulated, while if log_2_fc ≤ 1.0 and *p-value* 0.05, the corresponding gene was considered to be downregulated during various abiotic stresses. Amongst the 22 genes studied, we found eight of them viz. *D3*, *LRK1*, *OsEATB*, *RCN1, LOG*, *DEP1*, *SP1*, and OsSPL14, to be significantly regulated by different abiotic stresses (Figure3). The expression profiles of this set of stress-responsive genes were re-validated using qRT-PCR. For expression analysis via qRT-PCR, we used a moderately stress-sensitive rice cultivar IR64. For most of the genes analyzed here, the expression patterns obtained from this analysis were found to be similar to that in microarray (Figure4). The lack of correlation between the expression patterns in microarray and qRT-PCR in case of few genes in particular conditions, for example *LOG* in heat, *LRK1* in drought etc., can be attributed to relatively lesser specificity of probes in microarray and array-platform bias (Lee et al. 2007). Nevertheless, expression patterns obtained via qRT-PCR confirmed altered expression of these genes under various abiotic stress conditions.

In our qRT-PCR based analysis, *D3* – a negative regulator of tillering, was found to be significantly upregulated in cold, drought, and heat stresses (Figure4A) while *LRK1* – a positive regulator of tillering, had lower expression levels under salinity and high temperature conditions as compared to control (Figure4B). This finding may provide a basis for low number of tillers observed during various stresses. Besides, *OsEATB* – a negative regulator of plant height was significantly upregulated in all the analyzed stresses (Figure4C). Furthermore, in our analysis *OsSPL14* was upregulated under cold while *RCN1* was downregulated under cold, salinity, and heat stress conditions (Figure4D, E). This observation partially explains one of the mechanisms used by the plant for completion of its life cycle earlier during stress conditions; as *OsSPL14* promotes panicle branching (Miura et al. 2010) while *RCN1* negatively regulates vegetative to reproductive phase transition (Nakagawa et al. 2002). Moreover, three of the genes controlling the rate of spikelet formation viz. *LOG*, *DEP1*, and *SP1* showed down regulation in response to most of the stresses (Figure4F-H). This down regulation of gene expression may partly be responsible for smaller and less-branched panicles under stress conditions.

### Presence of putative stress-related *cis*-elements in the upstream region of stress-regulated ‘yield-related’ genes

**Figure 5:**
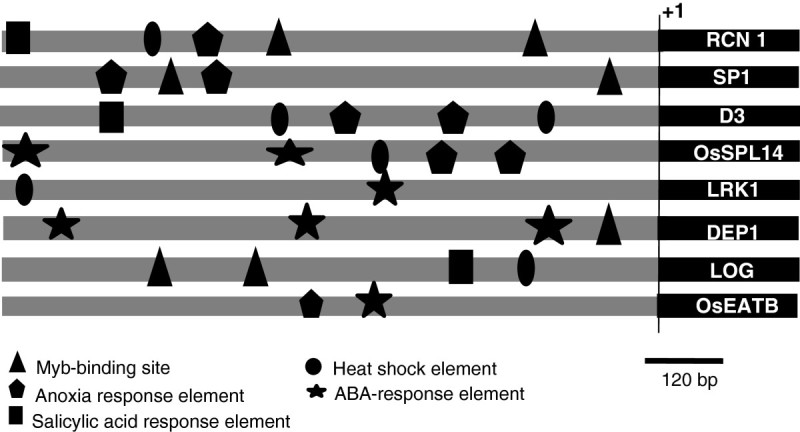
***In silico*** **analysis of the putative promoter region of stress-regulated ‘yield-related’ genes of rice.** Diagram shows the approximate positions of putative stress-related cis-regulatory elements present in the ~1 kb upstream region of various stress-regulated ‘yield’ genes of rice as predicted by PlantCARE database. Various stress-related elements viz. Myb-binding site (MBS), Anoxia response element (ARE), Salicylic acid response element (SARE), Heat shock element (HSE), and ABA-response elements (ABRE) are represented by different shapes as depicted above.

In order to comment upon the basis of stress-regulation of the aforesaid yield-related genes, we analyzed their upstream region for the presence of stress-related *cis*-elements. For this, the sequence corresponding to ~1 kb upstream region was retrieved for each of the eight genes and the sequence was analyzed for the presence of such elements using the database Plant CARE (Lescot et al. 2002). Several of the known stress-related *cis*-elements such as heat shock element (HSE), MYB-binding site (MBS), Anoxia-response element (ARE), ABA-response element (ABRE), and salicylic acid response element (SARE), were predicted to be present therein (Figure5). The presence of such elements elucidates regulated expression of the corresponding genes during different stress conditions, which remains to be confirmed experimentally.

## Discussion

Higher yield is a major target of crop improvement programs in rice due to obvious reasons. The agronomically favored traits include a higher number of tillers, larger panicles, more number of spikelets, proper duration of panicle differentiation, longer and slender grains, optimum grain filling and shorter plant height (Sakamoto and Matsuoka, 2008; Xing and Zhang, 2010). Besides, an optimal source-sink balance is also required to maximize yield in crop plants (Reynolds et al. 2009). Crop improvement aiming incorporation of all the favored traits requires profound understanding of the molecular basis of these traits.

Yield components are generally considered as a suite of complex traits controlled by multiple genes each with small effects. Previous studies comprising mutant analyses, comparative genomics, and map based cloning of QTLs have led to the identification of numerous genes (Table1), needed for the basic developmental processes affecting the development of tillers and panicles, as well as genes for quantitative changes of the traits such as number and size of grains and panicles, and plant height (reviewed by Xing and Zhang, 2010). In our classification based on the predicted molecular function of the genes (Figure1), the maximum fraction (30%) comprised transcription factors. Considering the capacity of transcription factors to affect expression of a wide variety of cellular targets, it is not surprising that a significant proportion of the genes regulating this highly complex trait are transcription factors. Besides, the next higher proportion of proteins involved in signal transduction corresponds to the importance of various converging and diverging signaling pathways operative during plant development.

### Developmental expression profile of yield-related genes in rice

One of the major factors which contribute to the grain yield, is the level of expression of different classes of yield-related genes which, in turn, is determined prima-rily by transcriptional regulation. Although several other factors including regulation at the translational or post-translational level also contribute to determine the final activity of an encoded protein, nevertheless, transcription is a major point of regulation of gene-expression. It has been found in several previous studies that transcript profiles of various genes often correspond to their physiological and developmental function (Ashikari et al. 2005; Kumar et al. 2012; Singh et al. 2012).

Altering the expression of individual genes has been shown to improve grain yield in rice (Xing and Zhang, 2010). However, considering the complexity of yield traits, yet more desirable results can be achieved by concurrent engineering of multiple genes (Naqvi et al. 2010). To achieve this objective via breeding or transgenic approaches, it is often necessary to select such genes which can function in a combinatorial fashion. Moreover, in transgenic approaches, it is appropriate to over-express or knock-down particular candidate genes only at certain developmental stage(s) which pre-requisitely requires information about developmental expression patterns and developmental stage-specific promoters. In this context, we first classified all the yield-related genes on the basis of the sub-component yield traits regulated by them (Table1). We then analyzed their expression during major stages of rice development viz. germination, seedling, tillering, stem elongation, booting, heading, flowering, milk, and dough (Figure2, Additional file1).

One of the central component traits determining the overall yield is the grain number per panicle. This trait is directly affected by the spikelet number, which further depends on the duration of panicle differentiation and rate of spikelet formation. We found the expression of one of the predicted positive regulators of panicle branching viz. *LAX1* to be higher in booting stage (Figure2A) thus showing a correlation between expression pattern and predicted developmental function. An earlier study by Oikawa and Kyozuka (2009) has shown that in *LAX1* mutants, the proliferation of meristematic cells is initiated but does not progress into the formation of axillary meristem. Similarly, in our analysis the other positive regulator *OsSPL14* had its highest expression at the heading stage (Figure2A). This finding is in agreement with an earlier report (Miura et al. 2010) which suggests that *OsSPL14* functions to promote branching in young panicles.

The rate of spikelet formation is regulated by genes controlling cell proliferation and thus affecting meristem size. This rate is critical in determining panicle size and spikelet number. In our analysis, we found higher levels of *DEP1* transcript until the heading stage (Figure2B). This indicates that *DEP1* affects spikelet number by enhancing meristematic activity and promoting cell proliferation as predicted earlier (Huang et al. 2009). Another gene known to affect rate of spikelet formation and hence grain productivity is a negative regulator – *OsCKX2*. It was identified by Ashikari et al. (2005) as a QTL derived from natural allelic variations, by comparison between a high-yielding indica rice variety and a low-yielding japonica rice variety. Our finding, that *OsCKX2* is expressed at a very low level throughout the development except for the flowering stage in elite indica rice varieties, further suggests that low expression of *OsCKX2* is required for higher number of spikelets and hence higher number of grains. The other gene regulating the rate of spikelet formation – *LOG*, had a high expression in all the developmental stages analyzed and interestingly, we found a negative correlation between the expression patterns of both these genes (Figure2B). As both of these genes are known to be part of cytokinin metabolism (Ashikari et al. 2005; Kurakawa et al. 2007), our results suggest that they play antagonistic roles in this important hormonal metabolic pathway.

The period from the first bracket primordium to heading is considered to be the duration of panicle development (Huang et al. 2006). Thus, this process is critical for the development of higher number of flowers and proper seed setting. Developmental expression profile, obtained via our analysis, for *RCN1* and *RFL* (Figure2C) suggests that *RCN1* co-ordinates panicle development and flowering time; while RFL plays a role in transition between vegetative to reproductive phases. Indeed, *RFL* is a rice ortholog of the *Arabidopsis* transcription factor *LFY,* which is known to regulate the transition of main growth axis to inflorescence (Rao et al. 2008).

Another developmental trait determining the number of panicles per plant and hence the number of grains is the tillering ability. Tillers are produced by shoot branching and is a two-stage process comprising the formation of an axillary bud at each leaf axil and its subsequent outgrowth. Grain yield is majorly contributed by the primary and early secondary tillers while late secondary and tertiary tillers make modest contribution even though they do consume nutrients and photosynthates (Xing and Zhang, 2010). Among the genes reported to regulate tillering, the *LRK1* expression profile strongly indicated that *LRK1* functions during tillering to increase the branch number (Figure2D). In an earlier study (Zha et al. 2009), it has been shown that over-expression of rice *LRK1* gene increased number of panicles associated with higher number of tillers.

The parameters determining grain weight are the volume and filling of the grain. Grain size is also a quality trait of the rice grain because long and slender grains are generally preferred. It is interesting to note that while most of the other yield traits are majorly regulated by positive regulators, this trait is mainly subjected to negative regulation. In our analysis the negative regulator of grain width, *GW2*, was highly expressed at milk and dough stages (Figure2E). A previous report by (Song et al. 2007) described that loss of *GW2* function increased cell number resulting in wider spikelet hull. Our data thus explain ‘slender’ grains found in many cultivated indica rice genotypes. Further, we found the highest expression of the positive regulator of grain weight – GIF1, in the milk and dough stages. This finding further supports its predicted role in promoting the formation of larger grains (Wang et al. 2008).

An increase in plant height makes plants more susceptible to ‘lodging’ which often leads to dramatic yield losses (Khush, 1999). Also, shorter plant height enhances the harvest index – the ratio of (grain) to (grain plus straw), thus increasing biomass production (Sakamoto and Matsuoka, 2008). Besides, semi-dwarf plants often show the ‘erect leaf phenotype’, which is advantageous for light capture (Sakamoto et al. 2006). Plant height is a complex interplay of action of different plant hormones and two of the known genes, action of which determines plant height, viz. *Sd1* and *OsBRI1* are involved in hormone metabolism (Sasaki et al. 2002; Morinaka et al. 2006). We found a strong correlation between the requirement of hormone action at certain developmental stages and the expression patterns of the respective genes (Figure2F). Furthermore, the expression profile of *OsBRI1* suggested that it may be one of the key players in the usually observed ‘tall’ phenotype.

### Expression profiling of yield-related genes under abiotic stress conditions

The growth and productivity of plants is greatly affected by environmental stresses such as temperature extremes, salinity, less water availability etc. One of the most drastic effects of these stresses is severe loss of yield potential, often referred to as yield penalty. While improper physiology and growth, and cell death are attributed to be the major cause of yield penalty during abiotic stresses, its molecular basis still remains enigmatic. Within our stringent parameters, we found eight of the genes, having a function in regulating yield, to be differentially regulated in response to one or more abiotic stress conditions (Figures3 and4). Out of these eight genes, *D3* and *LRK1* regulate tillering; *OsEATB* controls plant height; *OsSPL14* and *RCN1* are involved in panicle development and branching; and *LOG*, *DEP1*, and *SP1* regulate the rate of spikelet formation (Table1). Our *in silico* analysis of the promoters of these genes predicted the presence of several of the known stress-related *cis*-elements, thus attributing an apparent reason for their stress-induced differential regulation (Figure5). The expression profile of these genes (Figure4a-h) could explain different physiological observations under stress conditions such as reduced tillering, smaller and less-branched panicles, early completion of the life cycle due to shortened duration of vegetative and reproductive phases. All these can collectively lead to severe loss of yield.

In order to develop stress-tolerant varieties possessing satisfactory yield-potential even under environmental stress conditions, several factors need to be considered. Whilst our expression data show significant differential expression of some of the yield-related genes under different stress conditions, selecting the ‘best few’ requires certain physiological and developmental considerations. For instance, mutigene-engineering comprising stress-regulated ‘yield’ genes functioning in regulating tillering such as *LRK1* or *D3*, along with another regulating panicle branching such as *LOG*, might be beneficial taking into account the source-sink balance. An enhanced tillering ability may lead to relatively higher photosynthesis which, in turn, is a pre-requisite for bearing more spikelets. The advent of efficient rice transformation technologies (Hiei and Komari 2006; Nishimura et al. 2006; Sahoo et al. 2011) might help in paving the way for the development of such improved rice varieties via the transgenic approach.

## Conclusions

In order to achieve the objective of feeding the growing human population with diminishing arable land area, sustainable production of food grains is imperative. Our study provides some clues about the starting point of rice yield improvement via breeding and gene-pyramiding approaches. Furthermore, lesser yield during abiotic stress has often been observed even in stress-tolerant cultivars and our study predicts some targets that may be utilized to bridge this ‘yield gap’. Future studies may utilize the expression profile reported here, and investigate if over-expression or knocking-down of such yield-related genes can improve the grain yield under normal as well as stress conditions.

## Methods

### Sequence-retrieval and Microarray-based expression analysis of yield-related genes

Sequence of various yield-related genes (Table1) were retrieved from Rice Genome Database RGAP7 (Ouyang et al. 2007;http://rice.plantbiology.msu.edu/) using BLASTn and accession number given in the respective studies. The retrieved sequences were further validated by analyzing the annealing of primers used in the respective studies using Primer-BLAST (http://www.ncbi.nlm.nih.gov/tools/primer-blast/). The microarray probeset ids were retrieved using Rice Oligonucleotide Array Database (http://ricearray.org/; Jung et al. 2008). Out of the twenty-three yield-related genes reported so far, specific probe set ids could be found for all but one gene viz. *MOC1*.

For expression analysis, publicly available data for single microarray platform – 51 K Affymetrix gene chip was used since it covers most of the rice genes and the maximum microarray data in the public repositories has been generated using these chips. To study expression profile at various developmental stages, the normalized and curated signal intensities values on the 51 K array were retrieved using Genevestigator (Hruz et al. 2008; Zimmermann et al. 2008;https://www.genevestigator.com/gv/plant.jsp) at the stages representing germination, seedling, tillering, stem elongation, booting, heading, flowering, milk, and dough stage. Genevestigator is a reference expression database and was chosen as an analysis tool because it contains high quality manually assessed data (Grennan, 2006). To analyze the expression levels under different abiotic stress conditions viz. cold, drought, salinity and heat, the relative signal ratio values were retrieved using Genevestigator with default parameters (using experiments with id: OS00008 and OS00024) and the log_2_ transformed fold change values were calculated. Heat map with average linkage hierarchi-cal clustering was generated with Multi Experiment Viewer software (Saeed et al. 2006; http://www.tm4.org/mev/) using Pearson correlation as the distance metric.

### Plant material and stress treatment

Seeds of *Oryza sativa* L. cv IR64 rice variety were surface sterilized with 1% Bavistin and germinated in hydroponic system. Seedlings were supplied with modified Yoshida medium (Yoshida et al. 1972) and were grown under control conditions in growth chamber (SANYO, North America Corporation) at 28±2°C and 16 h/8 h photoperiod. The 10d old seedlings were subjected various stress treatments for 6 hours. For salt stress, the seedlings were shifted to Yoshida medium containing 200 mM NaCl; for drought stress, the seedlings were air-dried; for cold and heat stress, the seedlings were shifted to growth chamber at 4°C and 42°C respectively. Untreated seedlings were used as control.

### RNA isolation and qRT-PCR

Total RNA was isolated using TRIzol reagent (Life Technologies) from shoot tissues of both stressed and non-stressed seedlings as per the manufacturer’s protocol. RNA quality and integrity was analyzed using spectrophotometry and denaturing agarose gel electrophoresis. First strand cDNA synthesis was carried out from 2 μg of DNaseI-treated total RNA using RevertAid™ RNase H minus cDNA synthesis kit (Thermo Fisher Scientific Inc, USA) following the manufacturer’s protocol. Primers for real-time PCR were designed using Primer Express software v2.0 (Life Technologies, USA) either from the 3^′^-UTR regions (wherever possible) or the coding region of each of the genes (Table2). The primers were further validated for unique amplicon using Primer-BLAST (http://www.ncbi.nlm.nih.gov/tools/primer-blast/). qRT-PCR was carried out as described previously (Singh et al. 2012). The specificity of the amplification was tested by dissociation curve analysis and gel electrophoresis. Three technical replicates were analyzed for each sample. The relative expression ratio of each gene was calculated using delta C_T_ or comparative C_T_ value method (Livak and Schmittgen 2001) using *eEF-1α* as the endogenous control for normalization (Jain et al. 2006). The experiment was repeated for three biological replicates and the mean fold change was calculated and plotted along with corresponding standard deviation values.

**Table 2:** **Sequences of primers used for qRT-PCR**

Gene name	Primer name	Primer sequence (5’-3’)
*D3*	D3 Fw	TGCAGCCTTGTGGTTGCACCT
	D3 Re	TGGAAATCCACGGCCGCCAC
*LRK1*	LRK1 Fw	TACGCCCAGGCATGGGTTGC
	LRK1 Re	CGGAACTGGCCTCCTCCCAGT
*OsEATB*	OsEATB Fw	ATTGGCAGATGGGCGCGGAC
	OsEATB Re	CGCTGCACCGGAAAATGGCG
*OsSPL14*	OsSPL14 Fw	CGCAGACGCCTTGCAGGTCA
	OsSPL14 Re	ACCTGCGATGCTCACCAACAGA
*RCN1*	RCN1 Fw	TGCTGGTGGACAAACCCAACTGGT
	RCN1 Re	GTGGAGCCACCAAGCGACACC
*LOG*	LOG Fw	ACCGGCGGACGACGATACCT
	LOG Re	CGAGCTAGGGGCCGCCTTTG
*DEP1*	DEP1 Fw	CTGCGGATGCAACGGTTGTG
	DEP1 Re	TTTGCATTGGGCGCAAGAGC
*SP1*	SP1 Fw	TCTACTGGCTCCTCGCCGGG
	SP1 Re	CCGCCGCCTTCTCCTCCAAC
*eEF-1α* (endogenous control)	eEF-1α Fw	TTTCACTCTTGGTGTGAAGCAGAT
	eEF-1α Re	GACTTCCTTCACGATTTCATCGTAA

### *In silico* analysis of the putative *cis*-regulatory elements of the stress-responsive yield-related genes

Genomic sequences corresponding to the ~1 kb upstream regions of the respective stress-responsive yield-regulated genes were retrieved using chromosomal co-ordinates from the rice genome browser (http://rice.plantbiology.msu.edu/cgi-bin/gbrowse/rice/). The sequences were searched for the presence of putative *cis*-regulatory elements using PlantCARE database (http://bioinformatics.psb.ugent.be/webtools/plantcare/html/; Lescot et al. 2002).

## Electronic supplementary material

Additional file 1:**Microarray-based expression profile of yield-related genes of rice during various stages of development.** Scatter-plot shows microarray-based expression profile of various classes of yield-related genes of rice viz. genes controlling, (**A**) panicle development, (**B**) rate of spikelet formation, (**C**) duration of panicle differentiation, (**D**) tillering, (**E**) grain weight, and (**F**) plant height. The various developmental stages of rice have been shown on the X-axis. The values on the Y-axis represent transcript abundance (log_2_-transformed values of mean signal intensities on Affymetrix 51 K array) for each of the yield-related genes at the respective developmental stages. Error bars represent ±SE (standard error). (PDF 505 KB)

Below are the links to the authors’ original submitted files for images.

Authors’ original file for figure 1

Authors’ original file for figure 2

Authors’ original file for figure 3

Authors’ original file for figure 4

Authors’ original file for figure 5
