# Supplementary material for: Narrowing down the targets for yield improvement in rice under normal and abiotic stress conditions via expression profiling of yield-related genes
Source: Rice (N Y). 2012 Dec 22;5:37. doi: 10.1186/1939-8433-5-37 (PMC4883727; doi:10.1186/1939-8433-5-37)
Supplement: Supplementary file 1 — Additional file 1:Microarray-based expression profile of yield-related genes of rice during various stages of development. Scatter-plot shows microarray-based expression profile of various classes of yield-related genes of rice viz. genes controlling, (A) panicle development, (B) rate of spikelet formation, (C) duration of panicle differentiation, (D) tillering, (E) grain weight, and (F) plant height. The various developmental stages of rice have been shown on the X-axis. The values on the Y-axis represent transcript abundance (log2-transformed values of mean signal intensities on Affymetrix 51 K array) for each of the yield-related genes at the respective developmental stages. Error bars represent ±SE (standard error). (PDF 505 KB) [file 12284_2012_36_MOESM1_ESM.pdf]

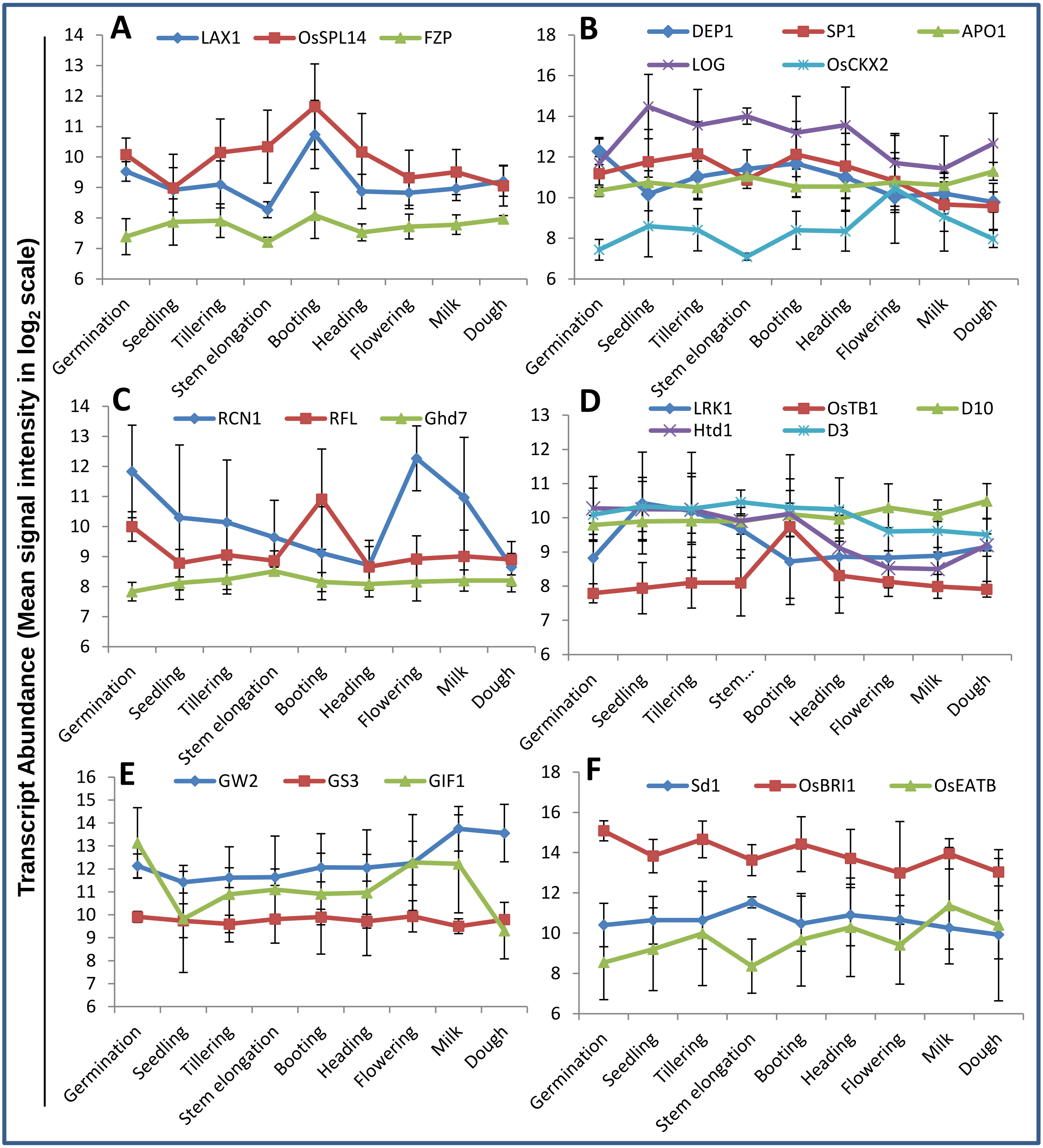

Additional File 1: Microarray-based expression profile of yield-related genes of rice during various stages of development. Scatter-plot shows microarray-based expression profile of various classes of yield-related genes viz. genes controlling - (A) panicle development, (B) rate of spikelet formation, (C) duration of panicle differentiation, (D) tillering, (E) grain weight, and (F) plant height. The various developmental stages of rice have been shown on the X-axis. The values on the Y-axis represent transcript abundance (log_2-transformed values of mean signal intensities on Affymetrix 51K array) for each of the yield-related genes at the respective developmental stages. Error bars represent ±SE (standard error).
