# Supplementary figures and images for: Narrowing down the targets for yield improvement in rice under normal and abiotic stress conditions via expression profiling of yield-related genes
Source: Rice (N Y). 2012 Dec 22;5:37. doi: 10.1186/1939-8433-5-37 (PMC4883727; doi:10.1186/1939-8433-5-37)

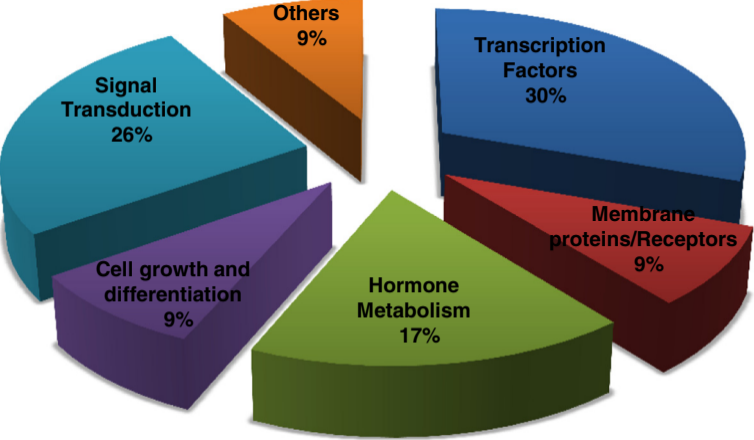

Supplement: Supplementary file 2 — Authors’ original file for figure 1 [file 12284_2012_36_MOESM2_ESM.pdf]

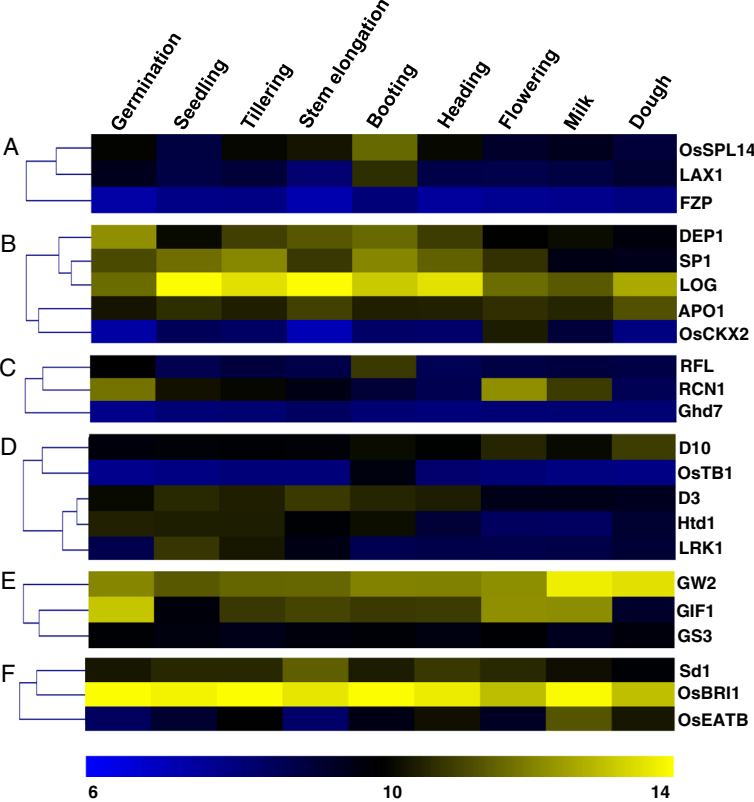

Supplement: Supplementary file 3 — Authors’ original file for figure 2 [file 12284_2012_36_MOESM3_ESM.pdf]

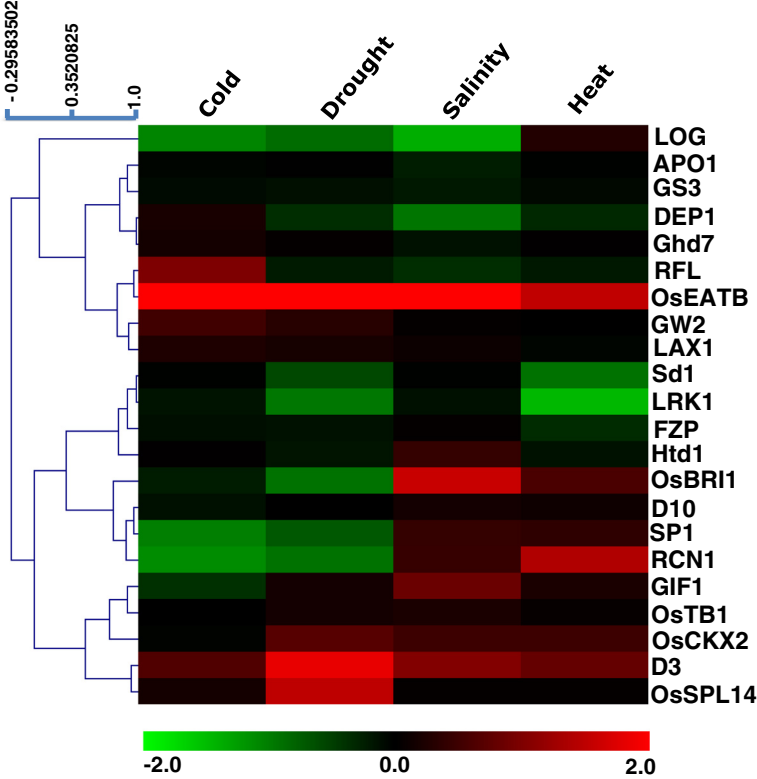

Supplement: Supplementary file 4 — Authors’ original file for figure 3 [file 12284_2012_36_MOESM4_ESM.pdf]

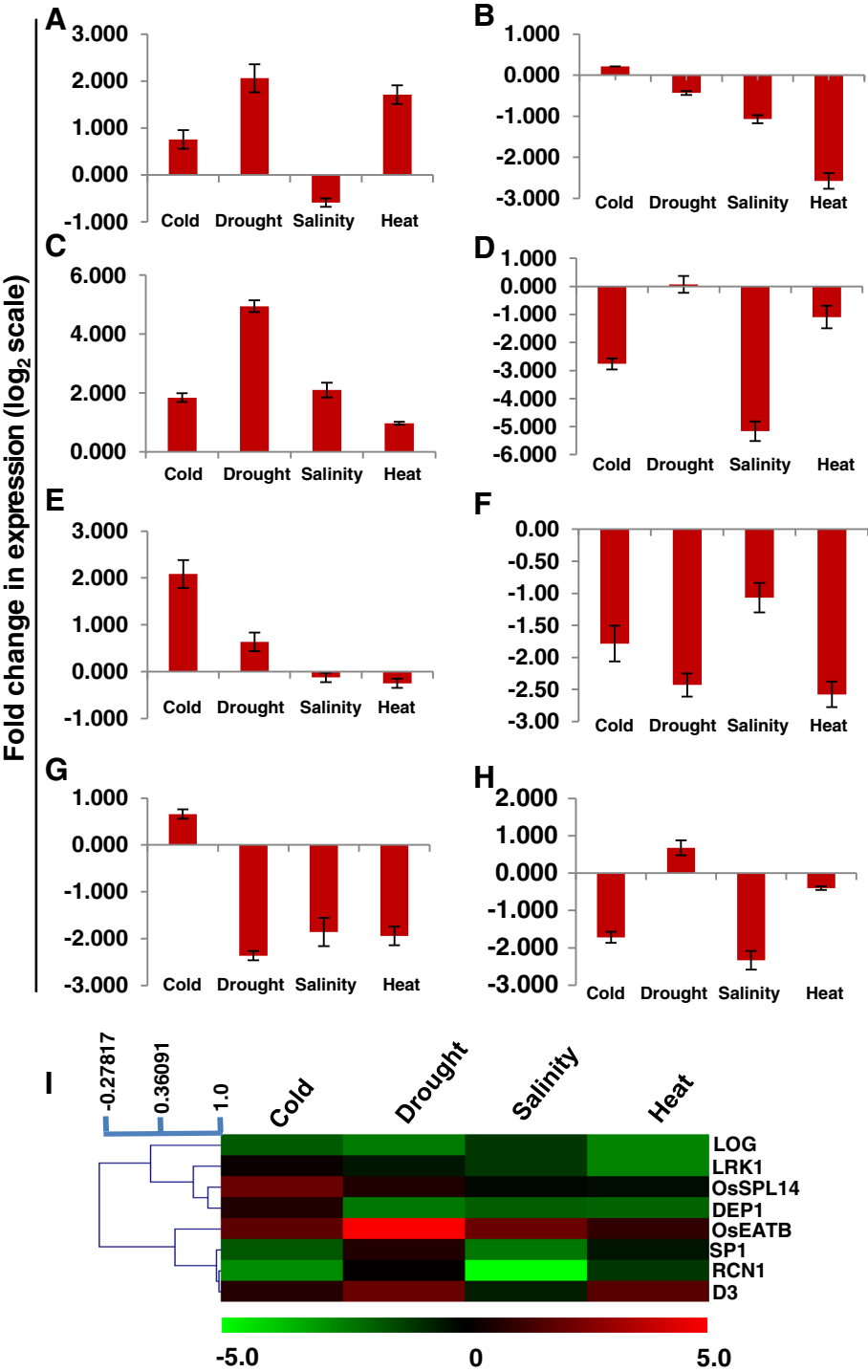

Supplement: Supplementary file 5 — Authors’ original file for figure 4 [file 12284_2012_36_MOESM5_ESM.pdf]
