# Supplementary material for: Narrowing down the targets for yield improvement in rice under normal and abiotic stress conditions via expression profiling of yield-related genes
Source: Rice (N Y). 2012 Dec 22;5:37. doi: 10.1186/1939-8433-5-37 (PMC4883727; doi:10.1186/1939-8433-5-37)
Supplement: Supplementary file 6 — Authors’ original file for figure 5 [file 12284_2012_36_MOESM6_ESM.pdf]

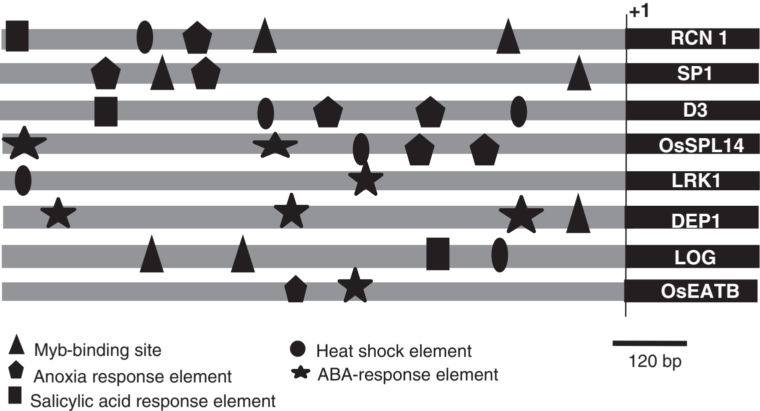
+1
RCN 1
SP1
D3
OsSPL14
LRK1
DEP1
LOG
OsEATB
Myb-binding site
Anoxia response element
Salicylic acid response element
Heat shock element
ABA-response element
120 bp
